# Exploring Medical Student Experiences of Trauma in the Emergency Department: Opportunities for Trauma-informed Medical Education

**DOI:** 10.5811/westjem.18498

**Published:** 2024-06-28

**Authors:** Giselle Appel, Ahmed T. Shahzad, Kestrel Reopelle, Stephen DiDonato, Frances Rusnack, Dimitrios Papanagnou

**Affiliations:** * Thomas Jefferson University, Sidney Kimmel Medical College, Philadelphia, Pennsylvania; † Icahn School of Medicine, Department of Emergency Medicine, New York, New York; ‡ Thomas Jefferson University, College of Nursing, Philadelphia, Pennsylvania; § New York University Grossman Long Island School of Medicine, Department of Emergency Medicine, New York, New York; ∥ Thomas Jefferson University, Sidney Kimmel Medical College, Department of Emergency Medicine, Philadelphia, Pennsylvania

## Abstract

**Purpose:**

During the third-year emergency medicine (EM) clerkship, medical students are immersed in traumatic incidents with their patients and clinical teams. Trauma-informed medical education (TIME) applies trauma-informed care (TIC) principles to help students manage trauma. We aimed to qualitatively describe the extent to which students perceived the six TIME domains as they navigated critical incidents during their EM clerkship.

**Methods:**

We employed a constructivist, modified grounded theory approach to explore medical students’ experiences. We used the critical incident technique to elicit narratives to better understand the six TIME domains as they naturally appear in the clerkship. Participants were asked to describe a traumatic incident they experienced during the clerkship, followed by the clerkship’s role in helping them manage the incident. Using the framework method, transcripts were analyzed 1) deductively by matching transcript excerpts to relevant TIME domains and 2) inductively by generating de novo themes to capture factors that affected students’ handling of trauma during critical incidents.

**Results:**

Twelve participants were enrolled and interviewed in July 2022. “Safety” was the most frequently described TIME domain, whereas “Gender, Cultural, and Historical issues” and “Peer Support” were discussed least. Inductive analysis revealed themes that hindered or supported their ability to manage traumatic experiences, which were grouped into three categories: 1) student interactions with the learning environment: complex social determinants of health, inequalities in care, and overt discrimination; 2) student interactions with patients: ethically ambiguous care, witnessing acute patient presentations, and reactivation of past trauma; and 3) student interactions with supervisors: power dynamics, invalidation of contributions, role-modeling, and student empowerment.

**Conclusion:**

The six TIME domains are represented in students’ perceptions of immediate, stressful critical incidents during their EM clerkship, with “Safety” being the most commonly described; however, the degree to which these domains are supported in students’ experiences of the EM clerkship differ, and instances of inadequately experienced domains may contribute to student distress. Understanding the EM clerkship through the specific lens of students’ experiences of trauma may be an effective strategy to guide curricular changes that promote a supportive learning environment for students in the emergency department.

Population Health Research Capsule What do we already know about this issue?

*Medical students are immersed in traumatic incidents when they rotate through the ED during the emergency medicine (EM) clerkship.*

What was the research question?

*To what extent do medical students perceive the domains of trauma-informed medical education (TIME) as they navigate the ED clinical environment?*

What was the major finding of the study?

*The degree to which TIME domains are supported in students’ experiences of trauma differ, and instances of inadequately experienced domains may contribute to student distress.*

How does this improve population health?

*Better identifying opportunities for trauma-informed education in the EM clerkship can support students and their well-being.*

## INTRODUCTION

Over recent years, emergency departments (ED) across the United States have served as points of care for victims of widening health disparities, increasing sociopolitical unrest, the COVID-19 virus, and increasing numbers of mass shootings. The ED workplace also serves as a clinical learning environment for learners across multiple health professions, routinely exposing them to trauma that is intrinsic to emergency medicine (EM) practice and care delivery in the US. Medical students are at high risk for sustaining psychological and emotional trauma from their encounters with patients and interactions with members of their ED care teams— often for the first time in their professional careers.^1^ ^–^ ^3^

Students have previously reported that their undergraduate medical education (UME) training has not adequately prepared them to cope with such stressful events.^2^ An inability to positively cope with stress can negatively impact students’ health, contribute to anxiety, depression, and/or detachment.^4^ ^–^ ^6^ Not only can this stress have a psychological impact on well-being, but it can also impact students’ ability to advocate for themselves, their peers, and their patients over the course of their training.

Research suggests that the impact of stressful events on students can be lessened or amplified by structural support embedded in formal curriculum. Frameworks, such as trauma-informed medical education (TIME), have outlined what structural support may look like in UME. Trauma-informed medical education represents an approach that applies trauma-informed care (TIC) principles to UME to help students recognize and address trauma in real time.^7^ It aims to create a learning culture guided by six trauma-informed domains: safety; trust and transparency; peer support; collaboration and mutuality; empowerment, voice, and choice; and cultural, historic, and gender considerations (Table 1).^8^

**Table 1.** Trauma-informed medical education domains.

Domain	Areas explored
Safety	Could the student express their thoughts about the situation openly? Did the student wonder if they would be negatively impacted if they voiced their opinion?
Trust and transparency	Was the student briefed about the situation before they experienced it?
Peer support	Were any of the student’s peers in the situation, too? How did the student interact with these peers?
Collaboration and mutuality	Was the student able to interact with their faculty? Did the student witness a lack of professionalism? What happened? How was this lack of professionalism responded to?
Empowerment, voice, and choice	If the student had feedback for your clerkship and/or other people, how did they deliver this feedback? If the student needed to take time away from the situation or clerkship, was there room in their schedule to do so? How could they request this time if they needed it?
Cultural, historic, and gender considerations	Were there others in this situation who had a similar racial, ethnic, or gender identity as the student? Were there others whose racial, ethnic, or gender identities were different from the student’s?

While some medical colleges have incorporated TIME domains into their curriculum,^9^ the extent to which TIME domains inform the third-year EM clerkship curriculum remains unclear and unexplored. By better understanding the student experience of the third-year EM clerkship, educators could take practical steps to adequately prepare learners for the challenges posed by the ED clinical learning environment. In this qualitative study, we aimed to describe specific TIME domains students experience as they are naturally immersed in the clinical environment. Specifically, we aimed to identify to what extent students perceived TIME domains to be present within the third-year EM clerkship curriculum to support their ability to navigate high-stress critical incidents in the ED.

## MATERIALS and METHODS

We took a modified constructivist grounded theory approach to collect, describe, and analyze third-year medical students’ perceived stressful incidents during their EM clerkship in their third-year of medical training. We employed the critical incident technique (CIT), a data collection method that guides participants to disclose vivid descriptions of a “critical incident.” Formally, per CIT a critical incident is defined as “any observable human activity that is sufficiently complete in itself to permit inferences and predictions to be made about the person performing the act.”^10^ As part of our CIT method, participants were asked to recount and describe a specific, stressful critical incident that they experienced during their EM clerkship. It is important to note that two study investigators have considerable experience with qualitative research methods (SD, DP), and one study investigator has extensive experience with the CIT method (DP). Also, one research team member (SD) is a trained trauma counselor with an extensive TIC background. The study protocol was approved by the institutional review board of Thomas Jefferson University, and data was collected in July 2022.

### Population and Sampling Strategy

Because we sought to understand the experiences of medical students in the EM clerkship, we intentionally and exclusively sampled third-year medical students. We recruited third-year medical students who had completed their EM clerkship at an urban, academic, tertiary-care medical center at Thomas Jefferson University Hospital in Philadelphia, Pennsylvania. Two study investigators (KR, DP) are faculty in this department and are familiar with the practice environment.

Student participants were identified through purposive convenience sampling. In July 2022, the study team sent recruitment emails to student listservs at our respective medical schools. Criteria for inclusion were that the participant was a third-year medical student and had successfully completed the required EM clerkship during the study period. Participants were enrolled through targeted emails describing the study and assurance that participation was voluntary. Study participants and their respective narratives were de-identified using an internally developed coding scheme.

### Data Collection

The study consisted of a series of critical incident interviews with third-year medical students, each 30–45 minutes long, that were conducted in August 2022. Informed consent was obtained from recruited students. After a brief demographic electronic questionnaire was completed, interviews were scheduled with participants. Each interview was conducted virtually using video conferencing software (Zoom Video Communications, Inc, San Jose, CA) with two members of the research team who were trained in eliciting critical incidents through interviews. Participants were compensated $75 for their participation in the study.

Interviews were conducted using a detailed interview protocol with questions that were informed by the TIME framework (Appendix). The interview protocol was developed by the study investigators and was piloted with two medical students to optimize interview questions. Pilot interview data were included in the analysis. Student participants were asked to describe a key incident during their EM clerkship when they experienced a significant trauma. An audio recording from each Zoom interview was transcribed using automated transcription software, which was then manually reviewed by members of the study team for transcription accuracy. Transcripts were de-identified.

### Analysis

Data for each participant’s transcript was reduced to capture a critical story. For each transcript, the participant’s narrative was re-organized chronologically into a coherent, re-storied narrative. By taking a constructivist and modified grounded theory approach, we inductively analyzed re-storied critical incidents. Through a series of several iterative conversations, and per CIT best practices, we manually developed assertions (organized in Excel [Microsoft Corp, Redmond WA]) about the meaning of each incident as it related to the research question. To organize overarching themes from assertions, we conducted a series of virtual card-sorting activities that were facilitated through several virtual Google Jamboard activities. Iterative cross-incident analyses with the study team informed connections, associations, and relationships for themes that emerged from the assertions. This allowed our team to generate an overarching framework of observed themes and relationships.

To ground our inductive findings and more directly assess the degree to which students’ perceived TIME domains during their clerkship, we undertook a deductive approach in which excerpts from each participant’s narrative were matched with the most relevant TIME domain discussed (ie, a vivid description of the impact the ED learning environment had on a student’s ability to openly express their opinions was matched to the TIME domain of “Safety”). For critical incidents where multiple TIME domains were identified, we selected the most dominant TIME domain. To ensure internal validity of data interpretation, the entire team of investigators met to review all inductive assertions and deductive categorizations. Discrepancies were resolved through iterative conversations over the 10-month study period.

## RESULTS

We identified and interviewed 12 student participants. Study size was informed by data saturation, information power, and analytical and data sufficiency.^11^ The average age of participants was 25.5 years old; 50% identified as male and 50% as female. The majority of participants identified as White (8 of 12 participants) (Table 2). Participants’ critical incidents aligned with deductive codes borrowed from the TIME framework (Table 3). Participants described elements of “Safety,” whether psychological or physical, most frequently across all 50 interviews, whereas “Gender, Cultural, and Historical issues” and “Peer Support” were discussed the least (13 each).

**Table 2.** Demographics of participants.

Characteristic	N = 12
Age	
21–24	4 (33%)
25–27	5 (42%)
27+	3 (25%)
Gender	
Man	6 (50%)
Woman	6 (50%)
Race	
Asian/Native Hawaiian/Pacific Islander	3 (25%)
White	8 (67%)
Hispanic	1 (8%)

**Table 3.** Frequency of deductive themes.

TIME domain	Representative quote(s)	Frequency table (as number of instances across all transcripts)	Percentage of all instances (%)
Safety	“I made an appointment with school counseling services. I felt fine. I kind of thought I was going to have, like, weird dreams or feel weird. And I was totally unaffected, but I don’t know. You never know. I don’t think I’ve ever seen someone…been there when they pronounce someone dead. It was kind of my first experience and even then, I was still periphery. [Counseling] was like, ‘I was going to reach out to you anyway. We made a list of those people who came up at the school counseling center’s meeting.’ I was feeling fine, so it wasn’t like, ‘Wow, that’s great!’; but it was kind of nice for someone to be, ‘It’s okay that you feel fine. You shouldn’t feel bad that you’re not, like, haunted by this,’ if that makes sense.”	51	34
Empowerment, voice, choice	“So I read the attending a little bit on edge, listening to what they’re saying, and then they say, ‘Yeah, like you didn’t even look into their mouth. And yeah, it just was not, not organized.’ And I was like in my head, like thinking, “Wait, what? Like I 100% did a neuro exam, and I had him open up his mouth and say, check for nine and ten. And then I had him point his tongue out to, to the right and left.” And then I was like, ‘Okay, what do I do now? Do I contradict my attending? Or just say, ‘Sure?’”	30	20
Collaboration and mutuality	“The physician brought the event up quickly. Once he was on the phone, consulted surgery and then hung up and he said, ‘It’s really important that we take ownership of what happened here. Instead of lying and/or making excuses, we take ownership and say what happened.’ But then he still had to go do other things, and then I was the one who initiated [more conversation about the event], which I think was appropriate from my sense and from the attending sense, a conversation about the event, mostly for a learning experience for me to talk about what happened.”	25	17
Transparency and trustworthiness	“I think it would have been nice to have some acknowledgment because like, part of the frustration is feeling like you’re the only person who sees it this way, you know? And it’s like, it would have been nice if my attending turned to me and was like, ‘Hey, like that was kind of problematic. I hope you don’t think that we all think that way because we don’t like that kind of thing.’ I think it would have counteracted a little bit of the disillusionment I feel towards medicine in general.”	18	12
Gender, cultural, and historical issues	“I think there’s always that feeling, especially like as a young female trainee, I feel like I kind of have to put on a brave face and not show that much emotion. I don’t know. Like it’s good to appear invested in your patients, but it’s not good to be like, ‘Oh, like this is the worst thing that’s ever happened, blah, blah, blah.’ Because obviously all these people have seen worse. So, no, I don’t think that anyone would have written me a bad review if I was showing that I was upset. But I do think it subconsciously impacts what people think of you. Like, you know, maybe she’s not cut out for this field or something.”	13	8.7
Peer support	“And so, I think that made me feel like I had to be the one responsible for ensuring that this woman was able to get home and avoid further intimate partner violence. I really felt like I was the one who decided, like, whether she would be undergoing more violence that night.”	13	8.7

*TIME*, trauma-informed medical education.

Analysis of critical incidents with cross-case comparisons revealed that themes were classified into three categories: 1) student interactions with the ED clinical learning environment; 2) student interactions with their supervisors; and 3) student interactions with their patients (Table 4).

**Table 4.** Framework of inductive themes.

Overarching grouping	Individual themes
Students’ interactions with patients	Reactivation of past trauma
	Emotional discomfort associated with navigating ethics and uncertainty
	Emotional discomfort when witnessing high acute patient care for the first time
Students’ interactions with supervisors	Power dynamics diminishing student clinical autonomy
	No validation for student clinical contributions
	Role-modeling as a means to support students
	Empowerment and feedback as a means to create psychological safety
Students’ interaction with the ED clinical learning environment	Navigating social determinants of health
	Responding to observed inequities in care
	Navigating race, gender, and socioeconomic discrimination

*ED*, emergency department.

### Student Interactions with the ED Clinical Learning Environment

#### Navigating Social Determinants of Health

Students struggled with having to navigate and reconcile the complex social determinants of health (SDoH) for patients seeking care in the ED. This was often accompanied by intense emotion and stress in students.

So, I obviously was a little stressed out because patients crying obviously is not something I have experienced…That’s just something that I hadn’t done on my own yet. And so being in the patient’s rooms with them crying and me just trying to be like, “What do I do? Do I want to fix it right away?” So I was like, “Maybe I should go get someone. Maybe he wants to talk about why he’s feeling this way.” I wasn’t really told how to deal with that.

Students experienced uncertainty when navigating overt patient suffering, psychosocial factors that further complicated care plans, and having conversations with their patients. Consequently, students desired more support during these encounters in the form of greater preparation for the patient encounter, emotional support, and effective role-modeling from their supervisors. They also expressed an interest in opportunities to debrief and make sense of these challenging encounters.

#### Responding to Observed Inequities in Care

Students experienced hesitation and distress when navigating instances of perceived care inequities with their treatment teams (eg, when the reason why two patients with similar presentations receiving different treatments was unclear).

And this patient looks exactly the same. …And I was sort of like, What’s the difference? They were like, “You could tell this one’s [the patient’s] uncomfortable.” I thought the other one looked uncomfortable, but whatever. We ended up doing just a lot more for her, and I couldn’t really identify any difference between the two cases. I thought they were both the same… “Hey, did that just happen, though?” I thought it happened. Or like, “Am I hallucinating?”

Inequity in care is a concept repeatedly introduced in the preclinical years; however, students felt unprepared to navigate discussions with their colleagues when they recognized inequity directly influencing the care they were delivering. Moreover, a third-year medical student’s relative lack of experience in the clinical realm and unfamiliarity with the unique environment of their rotation (eg, team dynamics, patient population, location), added further ambiguity to the situation. Students felt unable to communicate their concern and advocate for their patients.

#### Navigating Race, Gender, and Socioeconomic Discrimination

Discrimination (ie, racial, cultural, and/or gender-based) in the workplace hindered the psychological safety of students belonging to minority groups that were discriminated against—even when these acts were not directed to students (eg, at staff or patients belonging to the student’s minority group).

It’s also disappointing because I felt like my attending didn’t really say anything to go against that. He wasn’t agreeing with her, but he was like, “Oh yeah.” He kind of just dismissed it and didn’t engage too much.

A discriminatory experience coupled with silent complacency (ie, a lack of acknowledgment of such events from clinical team members, particularly faculty and supervisors who were tasked with advocating, teaching, and protecting students) complicated students’ responses to such events and marginalized their role in the clinical workplace. Importantly, students of color (as in the above case) felt less empowered on the team, especially when being their team’s only member of color.

### Student Interactions with Supervising Residents and Attendings

#### Power Dynamics Diminishing Student Clinical Autonomy

Power dynamics interfered with students’ ability to advocate for their patients and contributed to their stress when immersed in the clinical environment. These power dynamics hindered their ability to clarify complex medical and social cases because they feared being perceived as unknowledgeable. Furthermore, they were less likely to ask for a discussion and/or debriefing to resolve their feelings surrounding patient encounters as they did not want to be perceived as emotional or “weak”:

I felt sad and kind of disappointed that other physicians weren’t taking it seriously, that we needed to get her out of there and we could at least provide resources, or we could do more to acknowledge that she’s here for intimate partner violence-related concerns and that no one really cared about what happened after she left.

Students often found themselves torn between the desire to invest more time in caring for and advocating on behalf of their patients and the need to maintain efficiency, subordination, and a strong performance for evaluators who assessed their clinical skills, often overlooking unassessed yet equally vital interpersonal skills (ie, demonstrating empathy, advocating for patients).

And I didn’t really feel like I could explain to the physician, “Hey, this is something that is really important to me that I think should be handled differently.” I just didn’t feel that was my place with someone who had a lot of control over my grade. It was this war between what I feel was best for my future grades and what I felt was morally right.

#### No Validation for Student Clinical Contributions

A lack of acknowledgment of students’ contributions to the care team, either in real time or on their evaluation, caused students to experience distress. In complex clinical situations where students felt they had meaningfully contributed to patient care, a lack of validation of the student’s composure, adaptability, and/or judgment hindered their ability to assimilate to the care team and triggered dissociation from and disillusionment toward the clerkship experience.

You get an evaluation for every single stuff you work on. My EM evaluation didn’t say anything about [the mass shooting event]. It wasn’t like I needed anyone to be “Wow, she didn’t freak out” or “She was calm.” It didn’t have to be that. And my evaluation wasn’t bad, it was fair…Just to me, that’s kind of a large event that happened. It just felt kind of weird that that context was missing.

#### Role-Modeling to Support Students

Modeling of integrity, professionalism (ie, accountability), and debriefing by supervisors helped students process poor clinical performance and gain closure during iatrogenic complications.

The physician brought the event up quickly. Once [he[ hung up after talking with surgery, he said, “It’s really important that we take ownership of what happened here. Instead of lying and or making excuses, we take ownership.” Then he personally addressed my feelings and my potential blaming of myself for the situation and reassured that it wasn’t my fault. And these things he said, which is admirable, and how you take full, full ownership for the complication.

Participant narratives suggested that taking ownership over a stressful or traumatic incident offered a sense of agency and opened opportunities for positive clinical learning, instead of leaving the student feeling powerless to speak or act on the event. The negative impact of performing a clinical error was mitigated based on how next action steps were framed.

#### Empowerment and Feedback to Create Psychological Safety

As students worked through a variety of clinical encounters during their rotation, one factor that was often described as supporting students’ psychological safety was feeling empowered by their team and receiving timely feedback before, during, and/or after a clinical situation.

Them putting that trust in me…made me want to try to do the best that I could in this situation with a limited number of experiences.

In this particular incident, the student found themselves in an emergent encounter with a patient in cardiac arrest and was tasked with intubating the patient. Although the student felt uncertain, the team’s trust and empowerment to perform a critical role improved their sense of agency and security and helped motivate them to perform the task.

### Student Interactions with Patients

#### Reactivation of Past Trauma

Clinical situations involving patients who were victims of domestic violence reactivated trauma in students with similar experiences.

[Listening to her disclosure] was definitely hard because I’ve had not as extreme but similar experiences. I resonated with her, and I think that made it a lot more challenging. We could at least provide resources or do more to acknowledge that she’s here for intimate partner violence-related concerns. No one really cared about what happened after she left, or people were not even totally aware of it.

The clerkship curriculum did not prepare this student to manage the guilt associated with treating domestic abuse victims (or a sense of “not doing enough”). The student felt disappointed that other members of the clinical team were not acknowledging concerns for future domestic violence risk. Consequently, the student did not feel they could openly express themselves with their superiors who had control over their summative assessment (ie, grade).

#### Emotional Discomfort Associated with Navigating Ethics and Uncertainty

Ethically ambiguous situations abound in medicine. However, these ethically challenging situations—such as instances in which patients were physically/chemically restrained, procedures that were performed on patients with altered mental states, or critical medical decisions that were made by patient surrogates—caused students to experience emotional discomfort that made it difficult to navigate these situations in real time.

[The facial laceration] needed to be repaired. But the patient, understandably, who was confused really didn’t want us touching her and didn’t want it done… What we ended up doing is holding the patient down while the resident repaired the facial laceration. And during the entire time, she was obviously very unhappy, kept on telling us to stop, and told the resident that she would get her fired. We were able to successfully complete the procedure, which was our goal.

#### Emotional Discomfort Witnessing High-Acuity Patient Care for the First Time

The educational experience of the EM clerkship is primarily based on clinical encounters. However, witnessing acute manifestations of patients’ conditions, such as physical disfigurement or inability to communicate, overwhelmed students and impeded their ability to effectively manage the concerns of their patients. In the example below, while physical disfigurement was expected, the student felt stunted by a lack of preparation on how to navigate an encounter with a patient with alternative methods of communication and was overall unprepared for the effect that the patient’s physical disfigurement would have on their own composure.

I have never seen someone disabled for a while; I feel like we do have proper training on that. But then when I see, “Oh, my God, he wasn’t like this a tiny bit ago, and now he is because of disease. They used to be fully able and now they’re not.” I was trying to walk a line of the best way to communicate, which was a different experience than I ever had before. It was really hard to see a person in that state, especially when you’re not expecting it. I’ll remember what he was like forever.

## DISCUSSION

Student experiences explored in this study reinforce prior literature’s understandings of the ED as a stressful environment and the EM clerkship as an educational setting that may expose students to trauma that exceeds their existing coping abilities. The deductive and inductive analyses undertaken in this study reveal insights about the nature of these stressors through the lens of students’ experiences during their third-year EM clerkship. The deductive approach matched incidents shared within 12 students’ narratives to the six TIME domains, whereas the inductive approach of this qualitative data generated a novel group of 10 overarching themes.

Overall, the deductive analysis demonstrated that TIME domains were represented in students’ perceptions of EM clerkship educational structures (eg, mentors, policies, types of patient interaction) as they navigated critical incidents. However, there are differences in the frequency of and ways in which certain domains are represented and supported in the students’ perception of existing curricular structures. “Safety” was by far the most frequently matched TIME domain to excerpts within students’ narratives (34% of all identified instances), followed by “Empowerment, Voice, and Choice” and “Collaboration and Mutuality” (20% and 17% of instances, respectively).

In contrast, the TIME domains of “Transparency and Trustworthiness,” “Gender Cultural, and Historical Issues,” and “Peer Support” were less commonly identified as dominant domains across students’ critical incidents (12%, 8.7%, and 8.7% of instances, respectively). Excerpts that were matched to these TIME domains often described instances in which these domains were discussed but not positively reinforced in the current clerkship curriculum (Table 3). For example, an excerpt matched to “Safety” may have expressed an experience in which a student felt unable to voice their opinions openly due to perceived negative consequences. In contrast, other excerpts in student narratives reflected instances in which a particular TIME domain may have been well represented and supported by their clerkship experience. For instance, as described in Table 3, an excerpt that was matched to “Collaboration and Mutuality” demonstrated how this domain was supported by a supervisor’s role-modeling of professionalism.

Often, instances where TIME domains were positively reinforced resulted in stronger student resilience and handling of critical incidents, whereas the lack of positive reinforcement of TIME domains resulted in student distress. The prevalence of “Safety” as a domain identified across incidents (often as a domain that was not well supported) may indicate that students experience “Safety” as one of the most salient domains that affects their ability to manage stressful situations compared to other domains.

The inductive approach generated 10 de novo themes that describe students’ perceptions of trauma during the EM clerkship and can be broadly organized and understood as emerging from three types of student interactions: with the clinical environment; with supervisors; and with patients (Table 4). “Students’ interaction with the clinical learning environment” describe the immutable contextual environment of the clerkship experience (eg, social, racial, and/or cultural factors) that influence students’ interactions with others and includes navigating SDoH, acting on perceived inequities, and handling discrimination in the ED. In contrast, “Students’ interactions with supervisors” captures the themes of power dynamics, distress from invalidated contributions to the team, role-modeling, feedback, and empowerment.

Finally, the third category, “Students’ interactions with patients,” encompasses themes of emotional discomfort with ethical ambiguity, emotional distress from acute patient care conditions, and reactivation of previous personal trauma. Overall, these three families of themes paint a picture of the third-year EM clerkship in which students perceive themselves to be at the center of the complex learning environment of the ED, continuously interfacing with patients and supervisors in a pre-existing, fixed, socioeconomic, political, and cultural context that shapes the learning experience. The 10 themes that describe this complex setting have potential to either “enable” or “disable” effective student management of their critical incidents.

When comparing the inductively generated framework with the TIME framework in how they capture students’ experiences of the third-year EM clerkship, both frameworks demonstrate a considerable degree of overlap but differ in how they categorize students’ responses to critical incidents. Both frameworks describe the influence of power structures, debriefs, transparency of expectations, gender and cultural issues on students’ experiences. However, where the TIME framework categorizes these ideas based on the type of stressor, the inductive framework categorizes the same ideas based on whom the student was interacting with. Thus, one of the largest differences to emerge between the deductive and inductive analysis was not the content of themes but the overall principles by which the approaches organized and understood the content of student narratives.

We felt that the TIME framework yielded a broader, less specific picture of students’ experiences with critical incidents that were not as effective at capturing the nuances and complexities of students’ experiences and specific stressors (eg, SDoH, patient disfigurement, inequities in patient care, ethical dilemmas, lack of autonomy and validation) as the inductive framework. This discrepancy may stem from the fact that the TIME domains are not based specifically on the third-year EM clerkship but instead on general ideas found throughout UME. In addition, the TIME domains as envisioned by Brown et al draw on structures, such as institutional policies, counseling services, and self-care techniques, that extend beyond the structures (ie, mentors, patients) that students actively interact with when critical incidents occur.^7^

By comparison, because our findings originate entirely from student experiences during these incidents, this framework may be better able to capture a more detailed and student-informed understanding of the trauma-informed structures in the third-year EM clerkship that most significantly impact their ability to navigate acute stress. Accordingly, our observed themes may offer a more relevant lens to assess for educational and environmental structures specific to the third-year EM clerkship that hinder or promote students’ responses to critical incidents, while TIME domains may offer non-specific relevance in capturing experiences of trauma across various clerkships.

## LIMITATIONS

Several limitations of this study are worth noting. As this study was conducted as an interview series using convenience sampling at one institution, the findings may not be fully generalizable. However, the findings of our study may serve as a foundation for future studies, which may be able to better assess the generalizability of their findings in the context of this study’s. Our limited sample size raises concerns about the extent to which our findings are generalizable across EM clerkships in the US or to a broader population of medical students across various geographic locations.

Some degree of recall bias also likely affected our study as participants were more likely to remember the most salient elements, positive or negative, of their experiences in the ED while omitting descriptions of other factors that aided or impeded their ability to manage the situation. We attempted to limit the effect of recall bias by using a critical incident technique-guided questionnaire that asked participants to actively recall as many elements of the situation as possible (eg, location, time of day, surroundings, involved people, etc) to avoid omission. Also, the degree of safety that participants felt in disclosing their true feelings to the interviewer may have affected disclosure of traumatic incidents across our participant pool, which might have been reduced by a neutral, third-party interviewer not affiliated with our academic institution. Future studies might employ a cross-sectional qualitative approach in which representative sites across the country can be sampled.

## CONCLUSION

At present, students experience all six TIME domains during their third-year EM clerkship, with some domains experienced more than others. The domains of “Safety,” “Empowerment, Voice, and Choice,” and “Collaboration and Mutuality” are frequently described in student accounts of responding to immediate, stressful critical incidents during the clerkship. The inadequate perception of certain TIME domains in EM undergraduate medical education during critical incidents may contribute to student distress and hinder students’ ability to respond to their perceived trauma. Considering the findings laid forth by this study, the specific nature of students’ experiences of trauma during the EM clerkship may be an effective tool to guide curricular changes that improve students’ ability to manage immediate stressors in the clinical environment.

## Supplementary Information



