# Supplementary material for: Exploring Medical Student Experiences of Trauma in the Emergency Department: Opportunities for Trauma-informed Medical Education
Source: West J Emerg Med. 2024 Jun 28;25(5):828–37. doi: 10.5811/westjem.18498 (PMC11418875; doi:10.5811/westjem.18498)
Supplement: Supplementary file 1 [file wjem-25-828-s001.docx]

**Interview Guide for Interviewers**

**Trauma-Informed Medical Education Within the EM Clerkship**

Remember to focus on the goal:

Remember to frequently ask: “*Could you tell me more about that? . . . .*”

Ask how they “responded,” not “coped.”

Hi, thanks for joining me today for this interview. Just to give you some information on this interview before we begin:

The purpose of this study is to describe the educational environment of the emergency-medicine clerkship. The interview will last around 30-45 minutes. This meeting will be recorded and transcribed for data collection, but all of your responses are confidential. We will de-identify any individuals including you, the interviewee, mentioned during the course of the interview. However, we cannot guarantee confidentiality if significant misconduct is described during the course of the interview, or if there is clear evidence that there was harm to anyone or yourself. Do you have any questions? You can let me know if you have any questions at any point of this interview. If it is fine with you, we will begin the interview now.

I am now going to start the recording.

**Prompt 1:** **I want you to start by** **thinking about your emergency-medicine (EM) clerkship. Now think about an event during your EM clerkship that was stressful or that had a significant emotional impact on you. For example, this may be a stressful situation that you or a patient experienced in the emergency department during your EM clerkship.**

**Let’s take a moment to think about that event, and whenever you’re ready, let me know.**

**Now, I will begin to ask you some questions. Please answer them as they might relate to or result from experiences in your EM clerkship.**

**About The Event Itself**

1. **What happened?**
   1. Can you start by telling me what happened, almost as if the event were a play? So by that I mean: set the scene, describe who was there, what was happening when you came in, and how the events unfolded
      1. After they share, say “Thank you for sharing that with me.”
2. **When was it?**
   - When did this occur during your clerkship?
   - Was this your first clerkship? If not, which clerkships did you have before this one?
3. Where was it?
   - Where were you?
     - Tell me briefly about the location you were working in.
     - Was there anything different or unique about the workplace or the shift?
4. **Who was involved?**
   - - Who were all the characters in the play?
     - In what ways were these individuals similar or different from you? Was anyone in the situation of similar or different gender or racial backgrounds than you?

- Were any of your peers in this situation, too?
  - - Were these peers new to you or peers that you had worked with before?
    - What were they doing?
- Were clerkship faculty members involved in the situation?
  - - What were they doing?
    - Were you able to access any leadership or faculty members if you needed them before, during, or after the event?
    - What were these people doing?
      - How did they “respond”?.
- Were there any interactions with individuals during the event that you felt negatively impacted your ability to handle the event?
- Did the other people in this situation impact how you felt about expressing your thoughts?
- What were you **feeling** in the moment? By that I mean: what were your emotions before, during, and after the event?

1. How did you feel that you should respond to the situation?
2. If you felt that you needed to leave the situation, were you able to do so?
3. What were you **thinking** in the moment? By that I mean: what were specific thoughts that crossed your mind before, during, and after the event?
   - Did you feel like you could express your thoughts and opinions about the situation openly?
     - If not, why?
     - Were you able to do anything about not being able to express your thoughts openly?
     - Who did you feel most comfortable sharing your thoughts/feelings with? Who did you not feel as comfortable sharing your thoughts with and why?
   - Did you wonder if you would be negatively impacted if you voiced your thoughts?
4. What **happened** in the end?
   - How did the event turn out? How did it resolve?
   - Was the incident acknowledged?
   - Was this incident discussed after it occurred?
     - If so, who discussed it and what did they say about the event?
     - Do you wish the event had been discussed after it happened? If so, how do you think it should have been discussed or reconciled?
   - Was this incident reconciled in any other way?
     - If so, how?
   - If you had feedback for your clerkship and/or other people, how did you deliver this feedback?
5. What do you think about this event **now**?
   - What changed in you?
   - Was there anything unique to this situation that caused you to respond differently than you normally would?
   - Did you feel that the clerkship prepared you to handle similar incidents in the future?
     - How do you think the clerkship could prepare you to handle similar incidents in the future?
6. Why was this incident **significant** to you?

**Opportunities Section**

1. Were there any resources or tools you needed during the event?
   - - Did you have them? If not, was there anywhere where you could access them?
     - Were any of these resources specific to the EM clerkship?
     - What resources/information/tools do you wish you had during this event

- What do you wish the faculty, your team members, peers, or others had done in this event?
- Did the clerkship curriculum prepare you to navigate this incident?
  - If yes, how?
  - If not, how do you think the curriculum should prepare you for this incident?
  - Did you learn things in other clerkships that might have helped you navigate this experience?

1. What do you wish was in place during this event that wasn’t?
   1. Faculty: what could the clerkship faculty have done with you or for you?

- What role do you think the clerkship faculty should have had in the event you described?
  - - - Do you wish the clerkship faculty members would have prompted you to talk about the event?
      - Would you have felt comfortable talking about the event at that moment?
  1. Did you escalate the event?
     1. Did you report this event?
     2. If yes/no, what factored into your decision?
  2. If you needed to take time away from the situation or clerkship, was there room in your schedule to do so?
     1. How could you request this time if you needed it?

1. Other player involvement: Was there anything you would have liked your others to have done in the situation?
   - - If peer were present: did your interactions with your peers impact your understanding and ability to manage the event?
2. Debriefing opportunity
   1. Did you interact with anyone after the event? Faculty, peers, teachers, friends, or family?
      - - How did your experience change after talking with this person/these people?
        - Was there anyone you wished you had interacted with after the event?
      - “And have you discussed this event/patient encounter with anyone since that shift, like even at home or in class/school, it could even be in the non-clinical environment?”

**Last Closing Questions:**

1. What else do you think I should know?
2. If I have any additional follow-up questions, may I please come back to you with them?
3. We are waiting to have the gift card prepared, but once they are ready they will be sent to you via your Jefferson email.

*References:*

- Brown, T., Berman, S., McDaniel, K., Radford, C., Mehta, P., Potter, J., & Hirsh, D. A. (2020). Trauma-informed medical education (TIME): advancing curricular content and educational context. *Academic Medicine*, *96*(5), 661-667. doi: <https://doi.org/10.1097/ACM.0000000000003587>
- Flanagan, J. C. (1954). The critical incident technique. *Psychological Bulletin, 51*(4), 327–358.<https://doi.org/10.1037/h0061470>
